# Molecular and Physiological Effects of Browning Agents on White Adipocytes from Bone Marrow Mesenchymal Stromal Cells

**DOI:** 10.3390/ijms232012151

**Published:** 2022-10-12

**Authors:** Girolamo Di Maio, Nicola Alessio, Gianfranco Peluso, Silverio Perrotta, Marcellino Monda, Giovanni Di Bernardo

**Affiliations:** 1 Human Physiology and Unit of Dietetic and Sports Medicine Section, Department of Experimental Medicine, School of Medicine, University of Campania Luigi Vanvitelli, 80138 Naples, Italy; 2 Biotechnology and Molecular Biology Section, Department of Experimental Medicine, School of Medicine, University of Campania Luigi Vanvitelli, 80138 Naples, Italy; 3 Institute Bioscience and BioResources, CNR, 80131 Naples, Italy; 4 Dipartimento della Donna, del Bambino e di Chirurgia Generale e Specialistica, School of Medicine, Università degli Studi della Campania Luigi Vanvitelli, 80131 Naples, Italy; 5 Center for Biotechnology, Sbarro Institute for Cancer Research and Molecular Medicine, Temple University, Philadelphia, PA 19122, USA

**Keywords:** mesenchymal stromal cells, adipogenesis, BAT, WAT, browning, irisin

## Abstract

Two different types of adipose depots can be observed in mammals: white adipose tissue (WAT) and brown adipose tissue (BAT). The primary role of WAT is to deposit surplus energy in the form of triglycerides, along with many metabolic and hormonal activities; as thermogenic tissue, BAT has the distinct characteristic of using energy and glucose consumption as a strategy to maintain the core body temperature. Under specific stimuli—such as exercise, cold exposure, and drug treatment—white adipocytes can utilize their extraordinary flexibility to transdifferentiate into brown-like cells, called beige adipocytes, thereby acquiring new morphological and physiological characteristics. For this reason, the process is identified as the ‘browning of WAT’. We evaluated the ability of some drugs, including GW501516, sildenafil, and rosiglitazone, to induce the browning process of adult white adipocytes obtained from differentiated mesenchymal stromal cells (MSCs). In addition, we broadened our investigation by evaluating the potential browning capacity of IRISIN, a myokine that is stimulated by muscular exercises. Our data indicate that IRISIN was effective in promoting the browning of white adipocytes, which acquire increased expression of UCP1, increased mitochondrial mass, and modification in metabolism, as suggested by an increase of mitochondrial oxygen consumption, primarily in presence of glucose as a nutrient. These promising browning agents represent an appealing focus in the therapeutic approaches to counteracting metabolic diseases and their associated obesity.

## 1. Introduction

Obesity and its associated metabolic disorders are major health challenges encountered by rich populations in the twenty-first century. Numerous multidisciplinary strategies have been proposed to counteract obesity, from changes in eating habits to more drastic approaches (such as endoscopic or bariatric surgery) with a common objective of weight reduction and subsequent alleviation of weight-related complications [1,2]. However, so far, no absolute pharmacological treatment has been identified for overweight and obese people.

Adaptive thermogenesis represents in some mammalians a valid strategy for heat production in the brown adipose tissue (BAT) depots [3].

Evolution has supplied humans and other placental mammals with BAT depots for heat production not associated with muscle activity (non-shivering thermogenesis) [4]. This activity appears considerable for human infants to preserve high values of inner body temperature [5].

Until a few years ago, the scientific community maintained that BAT depots were lost in adult humans. However, this idea was inverted a few decades ago when the presence of BAT depots was observed in adults, especially after cold exposure [6,7,8].

In recent years, numerous studies have demonstrated that the generation of BAT from white adipose tissue (WAT) using “browning agents” was able to stimulate energy expenditure and prevent obesity in mice [9,10]. It has been recognized that, in addition to the traditional brown adipocytes contained inside BAT depots, WAT contains some adipocytes that exhibit characteristics similar to brown adipocytes after specific environmental or drug stimuli (such as exercise, cold exposure, and drug treatment) [11]. These cells are referred to as beige or ‘brite’ (brown-in-white) adipocytes, and the phenomenon is consequently known as “browning” or “beiging” [12].

BAT thermogenesis depends on a great number of mitochondria and a high expression of UCP-1 (uncoupling protein 1) located on the mitochondrial inner membrane, which facilitates the uncoupling of fuel combustion (proton leak) and ATP production in order to expend energy as heat [13,14]. Thus, approaches that activate brown adipocytes in humans may possess important health consequences, particularly efficacious anti-obesity and antidiabetic properties [15].

Several pharmacological and plant-based browning agents are able to stimulate brown adipogenesis or beige cell induction under certain conditions (exercise, cold exposure, and in response to β-adrenergic agonists such as CL 316,243) [10,16,17,18]. Recently, we assessed the in vitro effects of some browning agents (GLP-1, rosiglitazone, GW501516, FGF-21, and Sildenafil) during white adipogenesis originating from mesenchymal stromal cells (MSCs), which are able to differentiate into WAT and BAT [19]. The MSCs contained in white adipose tissues/depots include a subpopulation: multipotent stem cells (differentiating into adipocytes, chondrocytes, and osteocytes), as well as stromal cells, fibroblasts, and progenitor cells [20,21].

In our previous study, we established that rosiglitazone and sildenafil were able to shift white adipocyte commitment/differentiation to a brown adipocyte phenotype [19]. This finding holds importance for understanding the molecular basis of obesity. In this context, these drugs are promising candidates for the therapeutic treatment of obesity. In any case, in fighting obesity, the trans-differentiation of mature white adipocytes into beige/brown cells is of paramount importance.

In this paper, our goal was to evaluate whether the previously analyzed drugs are able to induce the browning process in adult white adipocytes obtained from differentiated MSCs. We broadened our investigation by evaluating the potential browning capacity of irisin. Boström and colleagues demonstrated that muscular exercises induce a myokine synthesis called irisin that plays a crucial role in converting white adipocytes to brown adipocytes and in regulating energy expenditure. In particular, irisin works on WAT in vitro and in vivo through Pgc1α expression and stimulates Ucp1 protein expression until a large brown fat-like tissue is formed [10,22].

## 2. Results

MSCs were cultured in a white adipogenic differentiation medium for 15 days and then further incubated for 7 days with different drugs (GW, SID, ROS, and IRI).

The concentration of each drug was selected according to previous findings [23,24,25,26]. and the cytotoxicity test we performed to evaluate if the published range of concentrations could be used in our experimental model (Table 1).

**Figure 1:**
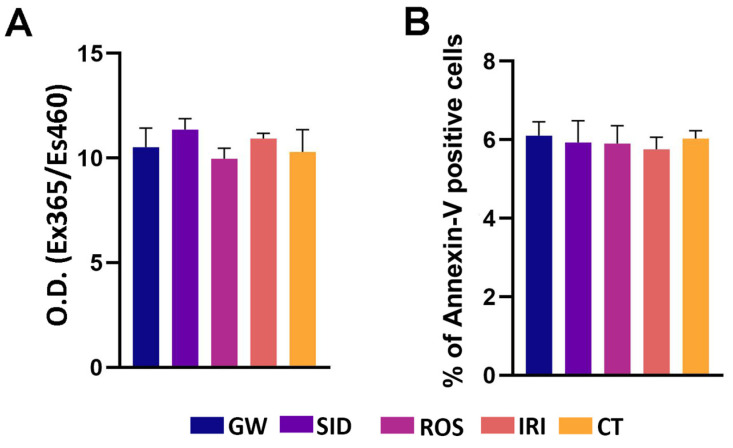
Senescence, and apoptosis detection. (**A**) Senescence evaluation in white adipocytes by 4-methylumbelliferyl-β-d-galactopyranoside (MUG) quantitative fluorescent assay following seven days of drug treatment. The 4-MUG is a β-galactosidase substrate that emits fluorescence when the enzyme converts it into fluorophore 4-methylumbelliferone. The fluorophore was detected with an excitation/emission wavelength of 365/460 nm. The data are expressed as arbitrary units (±SD, *n* = 3 biological replicates). The MUG assay detects the activity of senescence-associated beta-galactosidase (SA-β-gal) at pH 6.0, while, physiologically, beta-galactosidase is active at pH 4.0, the typical lysosomal acid environment. (**B**) Apoptosis analysis in white adipocytes by the fluorescein-conjugated Annexin V assay following seven days of drug treatment. The Annexin V assays detect the phosphatidylserine sites on the membrane surface of apoptotic cells by immunofluorescence staining. The graph shows mean expression values in cells treated with browning agents after white adipogenic differentiation (±SD, *n* = 3 biological replicates). GW: GW501516; SID: sildenafil; ROS: rosiglitazone; IRI: irisin; CT: differentiated white adipocytes without analyzed drugs. Drug concentration: 1 nM (GW); 1 nM (SID); 43 nM (ROS); 20 nM (IRI).

Then, we determined whether browning agents at selected working concentrations may affect key biological parameters by inducing phenomena affecting cell health (e.g., senescence, apoptosis). The senescence process was unaffected by the drugs we tested (Figure 1A); likewise, the apoptosis level was very low and did not change following incubation with drugs (Figure 1B).

**Figure 2:**
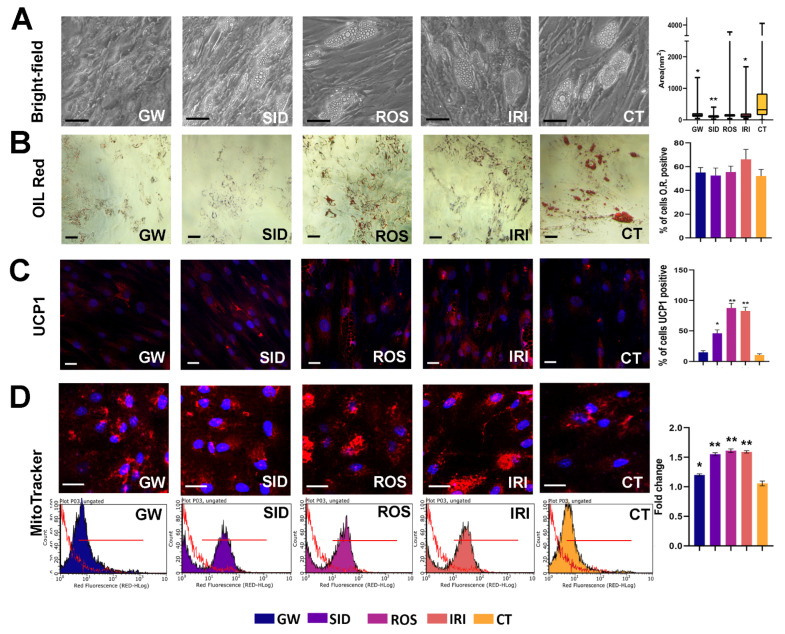
Lipid droplet size evaluation, Oil red O staining, UCP-1 expression, and MitoTracker analysis. (**A**,**B**): evaluation of lipid droplets by bright field analysis and Oil Red O staining in white adipocytes treated with browning agents for seven days. Scale bar: 50 µm. The right histogram of (**A**) shows the average droplet area in the different experimental conditions. For each cell, the size of lipid droplets was acquired with a CCD camera and analyzed with Quantity One 1-D analysis software (Bio-Rad Laboratories). We calculated the average droplet size, which was expressed in nm^2^. For every experimental condition, droplet size was determined by analyzing 100 cells. Data are expressed with standard deviation (*n* = 3, * *p* < 0.05, ** *p* < 0.01). The right histogram of (**B**) shows the percentage of Oil Red O positive cells (±SD, *n* = 3 biological replicates). (**C**): evaluation of UCP-1 expression detected in white adipocytes treated with browning agents for seven days. The UCP-1 level was detected in the cell cultures through antibodies against UCP-1 (Santa Cruz, TX, USA), according to the manufacturer’s instructions. The secondary antibody Alexa 594-conjugated was obtained from ImmunoReagents (Raleigh, NC, USA). Cell nuclei were stained with 4′,6-diamidino-2-phenylindole (DAPI) and then observed with a fluorescence microscope. The right histogram shows the percentage of UCP1-positive cells by counting a minimum of 500 cells in different microscope fields (±SD, *n* = 3 biological replicates; * *p* < 0.05, ** *p* < 0.01). (**D**): Analysis of mitochondrial mass in white adipocytes treated with browning agents for seven days. The micrograph showed the MitoTracker-stained mitochondria (red) in cells that were identified by DAPI nuclei staining (Blu). The graphs show the MitoTracker-stained cells acquired with Guava FACS Millipore Instruments; the red line corresponds to the unstained sample (negative control). The right histogram shows the intensity of MitoTracker staining as determined by FACS analysis. Data are reported as fold changes in intensity compared with control (white adipocytes) indicated as 1 (±SD, *n* = 3 biological replicates; * *p* < 0.05, ** *p* < 0.01). GW: GW501516; SID: sildenafil; ROS: rosiglitazone; IRI: irisin; CT: differentiated white adipocytes without analyzed drugs. Drug concentration: 1 nM (GW); 1 nM (SID); 43 nM (ROS); 20 nM (IRI).

Oil Red O staining is used to observe changes in the lipid metabolism of a cell and can validate white adipogenic differentiation by staining intracellular lipid droplets. In particular, the morphology of intracellular lipid droplets is large and unilocular in white adipose tissue but small and multilocular in brown adipose tissue. In our conditions, drugs did not induce a significant variation in lipid droplet staining (Figure 2B). Nevertheless, the GW, SID, and IRI treatments induced a significant reduction in lipid droplets size (Figure 2A).

Thermogenic adipocytes express high levels of UCP1 to dissipate energy (heat) by uncoupling the mitochondrial proton gradient from ATP production in mitochondrial respiration. We observed that SID, ROS, and IRI increased the percentage of UCP1-positive cells (Figure 2, middle panel). A small number of UCP1-positive cells was also detected in controls. This result is in concordance with studies showing that some brite adipocytes can be present in WAT [27,28].

The staining with MitoTracker, a red-fluorescent dye that stains mitochondria in live cells and whose accumulation is dependent on membrane potential, revealed that all browning agents increased the presence/proportion of mitochondria in these cells (Figure 2, lower panel). This result is in line with studies showing that during the differentiation of brown adipocytes there are qualitative and quantitative changes in the mitochondria [29,30,31].

We observed a significant increase in mitochondrial mass following drug treatments (right histogram in Figure 2).

**Figure 3:**
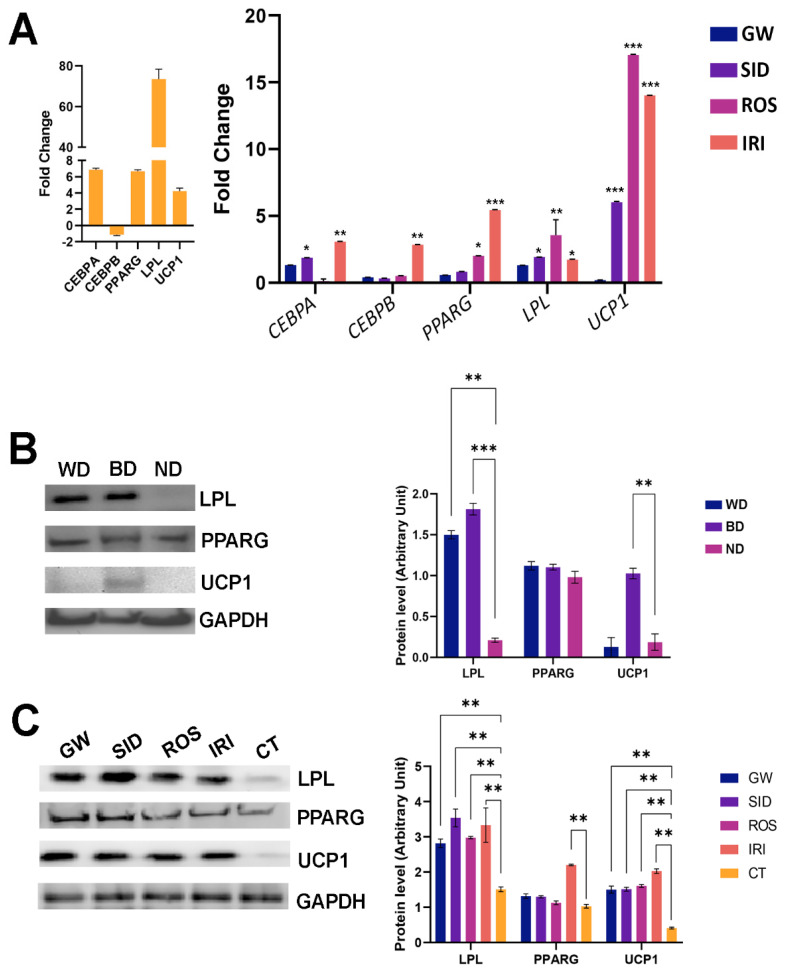
mRNA and protein levels of genes involved in adipocyte differentiation. (**A**): The left histogram shows the expression levels of adipocyte differentiation markers in MSCs following 21 days of incubation in white adipocytes differentiation medium. Data are reported as fold changes in mRNA levels compared to undifferentiated MSCs. The right histogram shows the mRNA levels in white adipocytes treated with browning agents for seven days compared with the control (untreated white adipocytes). Data are reported as fold changes in mRNA levels compared with control (white adipocytes) indicated as 1. Fold change values for genes were calculated as the ratio of the signal values of the experimental (drug-treated samples) group compared with the control group. (± SD, *n* = 3 biological replicates; * *p* < 0.05, ** *p* < 0.01, *** *p* < 0.001). (**B**): in vitro differentiation models. The protein levels of differentiation markers in MSC cultures following induction of white (WD) and brown (BD) adipocyte maturation, respectively. ND stands for control undifferentiated cultures. The right graph shows the expression levels of analyzed markers. Data are reported as arbitrary units compared with control (ND) (± SD, *n* = 3 biological replicates; ** *p* < 0.01, *** *p* < 0.001). (**C**): protein levels of differentiation and browning/thermogenic markers in white adipocytes (differentiated MSCs) that were treated with the above-reported drugs. CT stands for differentiation without drug supplementation. The graph shows the expression levels of analyzed markers. Data are reported as arbitrary units compared with control (CT) (±SD, *n* = 3 biological replicates; ** *p* < 0.01). GW: GW501516; SID: sildenafil; ROS: rosiglitazone; IRI: irisin. Drug concentration: 1 nM (GW); 1 nM (SID); 43 nM (ROS); 20 nM (IRI).

In our previous study, we identified the markers for white and brown MSC differentiation: white adipocytes showed high levels of CEBPA, PPARG, and LPL mRNAs, while brown adipocytes showed an increase in PPARG, LPL, and UCP1 mRNAs. This result indicates that the expression profiles of white and brown adipocytes contain both common (PPPARG, LPL) and specific markers, CEBPA and UCP1, respectively [19]. In this framework, our data reveal that SID, ROS, and IRI exhibited a browning effect by promoting strong UCP1 upregulation, while GW did not show a significant influence on UCP1 levels (Figure 3A).

We also observed an increase in early adipocyte markers (CEBPA and PPARG) following IRI treatment. This finding can probably be explained by the idea that IRI treatment could activate adipocyte differentiation of an MSC subpopulation remaining undifferentiated after stimulation with an adipocyte maturation “cocktail”. In support of this hypothesis, our observations in the same conditions also revealed an increase of Oil Red O-positive cells (histogram in upper panel, Figure 2).

We further validated our data with an evaluation of the protein expression of differentiation markers. As we already reported [19], the differentiation of MSCs in white adipocytes induced a significant increase of LPL along with a minimal upregulation of UCP1 and no modification of PPARG. This result indicates that the differentiation medium of MSC in white adipocytes also promoted the lineage specification of a subset of putative brown progenitors. (Figure 3B). The differentiation procedure to obtain brown adipocytes produced a strong increase in UCP1 protein levels along with LPL upregulation, as expected (Figure 3B). Globally these data validated our experimental model.

In the MSCs differentiated into white adipocytes and treated with the browning agents, we detected a strong increase in UCP1 and LPL protein levels (Figure 3C). These data are in agreement with mRNA analysis (Figure 3A).

Healthy cells can oxidize several substrates (carbohydrates, lipids, and proteins) for energy production (ATP) and can adapt nutrient oxidation to nutrient availability.

**Figure 4:**
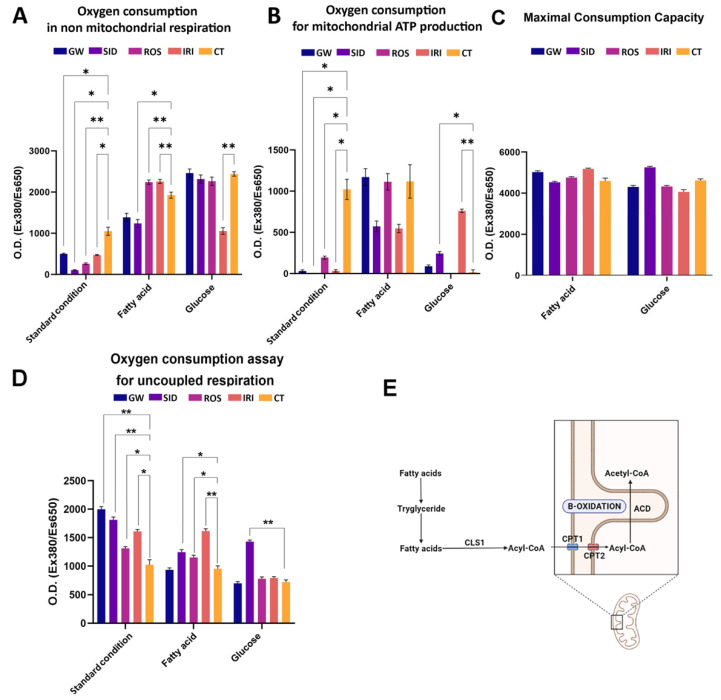
Evaluation of cellular mitochondrial function by oxygen consumption assay. (**A**): Basal oxygen consumption rate. Oxygen consumption rates (OCR) in non-mitochondrial respiration was measured by MitoXpress^®^ Xtra assay in white adipocytes treated with browning agents. Cells were incubated in complete medium (basal condition). The histogram shows optical density (O.D Ex380/Es650 nm) (±SD, *n* = 3 biological replicates; * *p* < 0.05, ** *p* < 0.01). (**B**): OCR for mitochondrial ATP production. (* *p* < 0.05, ** *p* < 0.01). (**C**): OCR for maximal respiratory capacity. (**D**): OCR for uncoupled respiration. OCR was measured according to protocol described in the methods section. Cells were incubated in complete medium (basal condition), or medium containing only palmitate (fatty acid), or medium containing only glucose (glucose). The histogram shows optical density (O.D Ex380/Es650 nm) (±SD, *n* = 3 biological replicates; * *p* < 0.05, ** *p* < 0.01). (**E**): A scheme to explain supposed futile lipogenic pathway with fatty acid and triglyceride formation, succeeded by triglyceride lysis and oxidation of free fatty acids. In particular, three enzymes, DGAT2, LIPIN1, and AGPAT2 generate triglycerides from fatty acids. These are subjected to the lysis in cytosolic lipid droplets by ATGL, HSL, and MGL. The released fatty acids can both activate UCP1 to produce heat or be transformed into acetyl-CoA by ACSL1 and shuttled into the mitochondria across the translocator CPT1and CPT2. Here they are submitted to beta-oxidation via ACD, to produce mitochondrial proton leaks (thermogenesis). GW: GW501516; SID: sildenafil; ROS: rosiglitazone; IRI: irisin; CT: differentiated white adipocytes without analyzed drugs. Drug concentration: 1 nM (GW); 1 nM (SID); 43 nM (ROS); 20 nM (IRI).

Cellular mitochondrial and non-mitochondrial metabolism were evaluated by determining oxygen consumption in white adipocytes treated with browning agents (Figure 4).

In white adipocytes, during lipolysis, free radicals such as reactive oxygen and nitrogen species are produced through a series of cytoplasmic activities—e.g., inside the endoplasmic reticulum, phagosomes, peroxisomes, and on the cellular membrane [32]. In most cases, all these reactions involve a large amount of non-mitochondrial oxygen consumption. In white adipocytes, a large oxygen consumption also occurs in mitochondria for ATP production. Brown adipocytes show less oxygen expenditure, since the production of cytoplasmic reactive oxygen and nitrogen species is reduced, and minimal oxygen is utilized for mitochondrial ATP synthesis [33]. Therefore, an effective browning stimulation entails a decrease in mitochondrial (Figure 4A) and non-mitochondrial oxygen consumption (Figure 4B), due to metabolic patterns that are typical of brown adipocytes. This result was obtained with all four drugs we evaluated (see standard condition histograms in Figure 4).

To gain more insight into these processes, we grew white adipocytes in media supplemented with a single nutrient (fatty acid or glucose) and then evaluated mitochondrial oxygen consumption, since it is well-known that thermogenic adipocytes (brown and beige cells) manage lipid and glucose homeostasis in order to modulate body temperature. Heat production, or thermogenesis, is a powerful energetic mechanism, and brown adipocytes need a fast and accessible power source, represented principally by glucose and/or free fatty acids [33,34]. Our results were consistent with the findings reported in the literature: in the presence of glucose, we observed an increase in mitochondrial oxygen consumption, primarily after treatment with GW, SID, and IRI (Figure 4B). In contrast, the sole presence of fatty acid in the media did not induce a significant variation of oxygen consumption among various treatments, compared with the control (Figure 4B).

The determination of maximal respiratory capacity (OCR in the presence of uncoupled oxidative phosphorylation, such as FCCP) indicates by what means cells react to an increased ATP demand. Generally speaking, the drug treatment preserved the respiratory capacity observed in the reference sample. This may suggest that no toxic effect due to drug treatment occurred. On the contrary, it should be underlined that in some conditions the maximal respiratory capacity was even increased compared with the controls (Figure 4C). Data on uncoupled respiration seems to confirm the beiging activity of analyzed drugs (Figure 4D). Indeed, uncoupled respiration is an important marker of a thermogenic adipocyte having increased uncoupled respiration due to UCP1. Notably, the increased uncoupled respiration was observed in standard conditions and in a medium supplemented with glucose.

Reports show that brown adipocytes uptake huge amounts of free fatty acids for a lipogenic futile cycle, which consists of the formation of triglycerides (TG) from free fatty acids and TG breakdown with beta-oxidation of released fatty acids (Figure 4E) [33].

**Figure 5:**
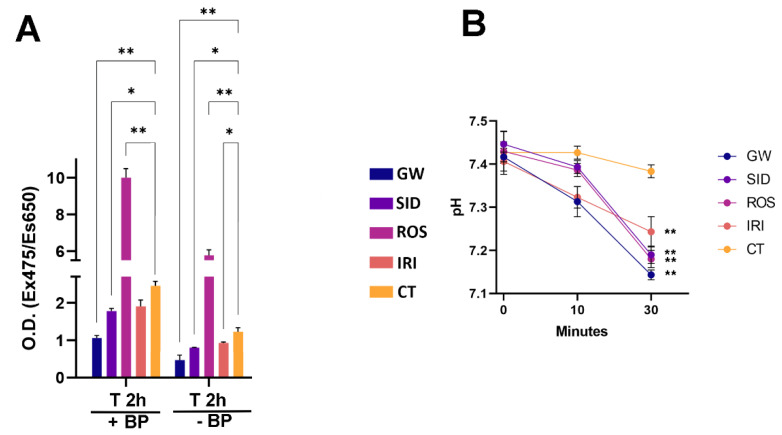
Uptake and release of FFAs in MSCs treated with browning agents after white adipogenic differentiation. (**A**): the cells were serum-starved for 16 h, then treated with 10 µM BODIPY FL C16 for 2 h in DMEM (T 2 h + BP). Subsequently, cultures were incubated for 2 h (T 2 h w/o BP) with complete medium (DMEM containing 10% FBS) without BODIPY FL CL16. The histogram shows the uptake levels of palmitate in different experimental conditions. (**B**): Cells were incubated in KREBS buffer with 2%BSA for 10 min, then the pH of the medium was evaluated at 10 and 30 min following the addition of 0.5 µg/mL epinephrine in the medium. Data are expressed as optical density (O.D Ex475/Es650 nm) (±SD, *n* = 3 biological replicates; * *p* < 0.05, ** *p* < 0.01). GW: GW501516; SID: sildenafil; ROS: rosiglitazone; IRI: irisin; CT: differentiated white adipocytes without analyzed drugs. Drug concentration: 1 nM (GW); 1 nM (SID); 43 nM (ROS); 20 nM (IRI).

In this scenario, we evaluated free fatty acids (FFA) uptake in white adipocytes at different time points following treatment with browning agents. In our experimental conditions, we observed increased uptake of FFA after treatments with ROS, which was able to stimulate and sustain browning (Figure 5A). We also evaluated the release of fatty acids following the stimulation of lipolysis by epinephrine treatment. We detected an increase in fatty acid release in samples treated with drugs compared with the control samples (Figure 5B).

## 3. Discussion

BAT is a key controller of total-body glucose and lipid homeostasis. Significant steps have been taken to better understand the molecular mechanisms of agents able to activate browning from WAT; this phenomenon appears useful for sustaining a growing energy consumption, avoiding body weight increase, and preventing obesity and related comorbidities (e.g., hepatic steatosis and type 2 diabetes) [35].

In our previous paper, we estimated the browning/beiging effects of some drugs through white adipose tissue differentiation of MSCs [19]. The drugs were supplemented at the beginning of the differentiation process and not on mature white adipocytes. Our finding was that GW, SID, and ROS were effective in promoting the shift of white adipocyte precursors to brown phenotype. This study proved the effectiveness of such drugs on adipogenesis, although it has limited value for obesity treatment. In this scenario, an effective drug must be active on pre-adipocytes and on white adipocytes by promoting their trans-differentiation into brown adipocytes. For this reason, we further extended our study on the effectiveness of the above-reported drugs in promoting trans-differentiation of pre-adipocytes and white adipocytes in brown adipocytes. In addition, our study also included the browning compound irisin (IRI), a myokine that is stimulated by muscle training and able to control energy expenditure.

In vivo studies (or even clinical studies) on some of the browning agents we evaluated in this study have already been reported [10,36,37,38]. In one of these studies, nanoparticles containing Rosiglitazone or CL 316243 charged into the transcutaneous microneedle patch formed by hyaluronic acid scaffold to locally induce brown adipocytes in white fat depots [39].

The browning induction of irisin in subcutaneous white depots in mice and humans have been plainly reported [22,40]. Although, the impacts of irisin on visceral fat remain under investigation.

We aimed to perform a comparative analysis to better evaluate the physiological and molecular effects of these agents in order to use them in a more rational way. The published papers used different experimental models and/or experimental conditions and hence it is difficult to perform a reliable comparison. Our paper aims to fill this gap.

An ideal experimental approach should be based on the use of adipocytes isolated from tissue (fat depots) [41]. Nevertheless, the in vitro cultivation of these mature cells is challenging and some experimental procedures we utilized cannot be performed in a reliable way. Many in vitro models for studies on adipose tissue differentiation and physiological performances rely on immortalized pre-adipocytes. These are useful models but present important limitations. Indeed, immortalized cells have an alteration of cell cycle regulation, and it is well known that definitive differentiation processes are strictly related to cell cycle status. In this scenario, data on the differentiation of immortalized pre-adipocytes may be unreliable. Furthermore, the differentiation process did not start from the pre-adipocyte phenotype; rather adipogenesis begins with lineage specification (commitment) of stem cells.

We decided to use a different experimental approach. We utilized human mesenchymal stromal cells induced to differentiate into white adipocytes. This procedure produces a heterogenous population: some cells become mature adipocytes, some others persist in a pre-adipocyte stage, and others are only lineage-committed. This may appear as a limitation, but it may reflect what is present in fat tissue depots. Indeed, in the fat depot, the majority of cells are mature adipocytes, but these depots contain a small percentage of pre-adipocytes, lineage-committed cells, and even stem cells [42,43]. In this scenario, our model has only one limitation: the percentage of mature adipocytes is reduced compared to white tissues; however, this will not affect the comparison study we performed.

Morphological analysis reveals that although the majority of browning agents did not induce a significant variation of lipid droplets, IRI treatments seemed to potentiate adipogenesis (Oil Red O staining). In addition, SID, ROS, and IRI treatments increased the percentage of UCP1-positive cells, which is typical of brown tissue. The effectiveness of shifting to brite/beige phenotype of white adipose cells also came from data on the uptake and release of fatty acids. It has been demonstrated that brown adipocytes actively uptake and release fatty acids [44]. Indeed, we evidenced that drug treatments of white adipocytes improved both the uptake and release of fatty acids. This result suggests that the treatments induced both morphological and functional changes in white adipocytes.

The above-reported findings agree with data showing increased mitochondrial mass after browning treatments. In addition, UCP1 mRNA expression levels also indicated that SID, ROS, and IRI had a browning effect.

The analysis of mitochondrial and cytoplasmic oxygen consumption in white adipocytes after browning treatments indicates that the drugs are effective in promoting a shift to a brite/beige metabolic profile with reduced oxygen expenditure. This hypothesis is strengthened by the data on mitochondrial oxygen consumption in the presence of a single nutrient. ROS and IRI were the most effective in utilizing glucose and in the uptake of free fatty acids for the lipogenic futile cycle.

In conclusion, in this study we demonstrated that a pharmacological approach could be envisaged for shifting white adipocytes into brite/beige adipocytes. The irisin treatment appears the most promising one since it is based on a natural polypeptide that is secreted from muscle cells. The next step in this research will be the in vivo evaluation of the effectiveness of such drugs. In detail, mice fed with a high-fat diet should be treated with irisin (and/or the other drugs we analyzed) to evaluate the effects on obesity.

## 4. Materials and Methods

### 4.1. MSC Cultures, in Vitro Adipocyte Differentiation, and Drug Treatments

Human MSCs were purchased (Lonza, Milan, Italy) and expanded according to the manufacturer’s recommendations. Cell cultures were seeded at a density of 1.5 × 10^4^ cells in six-well plates and grown in Low Glucose DMEM medium (EuroClone, Milan, Italy) containing 10% FBS (Euro-Clone, Italy) and 3 ng/mL of b-FGF (Prepotech, London, UK), 1% penicillin/streptomycin (Microgem, Naples, Italy), and 1% L-Glutamine (Microgem, Italy).

At 70–80% confluence, the medium was replaced with a white adipogenic induction medium, composed of high-glucose DMEM (Microgem, Italy) supplemented with 10% HS (Horse Serum) (Microgem, Italy), 1% penicillin/streptomycin (Microgem, Italy), 1 mM dexamethasone (Sigma-Aldrich, MO, USA), 10 µg/mL insulin (Sigma-Aldrich, Saint Louis, MO, USA), 0.5 mM 3-isobutyl-1-methylxanthine (Sigma-Aldrich, MO, USA), and 200 µM indomethacin (Sigma-Aldrich, MO, USA). Cells were cultured in this medium for two weeks, the media were replaced two times/week. After that, the cells were treated with different drugs—i.e., 1 nM GW501516 (GW) (Sigma-Aldrich, MO, USA), 1 nM sildenafil (SID) (Sigma-Aldrich, MO, USA) [45], or 43 nM Rosiglitazone (ROS) (Sigma-Aldrich, MO, USA)—or with 20 nM IRISIN (IRI) (Elabscience, Houston, TX, USA) [46], and they were incubated for another week. During this period, the media were twice changed, and drugs were re-supplemented.

Adipogenic differentiation was confirmed on day 21 using Oil Red O staining (Sigma-Aldrich, MO, USA).

For brown differentiation, the MSCs were cultured in the same condition as white adipogenic differentiation for 14 days. After that, the medium was changed to DMEM/F12 (EuroClone, Milan, Italy) and supplemented with 10% FBS, 200 uM of ascorbic acid (Sigma-Aldrich, Saint Louis, MO, USA), 20 nM of insulin, 0.2 nM of T3 (Sigma-Aldrich, Saint Louis, MO, USA) and 1 uM od b-adrenoreceptor agonist CL316243 (Sigma-Aldrich, Saint Louis, MO, USA). The cells were cultivated for another 7 days.

### 4.2. Cytotoxicity Assay

We plated 1000 cells per well in a 96-well plate and incubated them for 24 h with in-media high-glucose DMEM (Microgem, Italy) supplemented with 10% FBS (Microgem, Italy), and 1% penicillin/streptomycin (Microgem, Italy). Tested drugs were added to the media at different concentrations respectively. At the end of the incubation period, the toxicity was evaluated with Cytotoxicity Detection Kit (LDH) (Sigma-Aldrich, Saint Louis, MO, USA) following the manufacturer’s instructions. The untreated sample was the control (100% alive cells) and the percentage of live cells in drug-treated samples was calculated accordingly.

### 4.3. Senescence Assay (SA-Assay)

In the quantitative SA-β-gal assay, 4-Methylumbelliferyl β-D-galactopyranoside (4-MUG) (Sigma-Aldrich, MO, USA) was used as a substrate of β-galactosidase; 4-MUG does not fluoresce until cleaved by the enzyme to generate the fluorophore 4-methylumbelliferone. The assay was carried out on lysates obtained from cells treated with browning agents after white adipogenic differentiation. The production of the fluorophore was monitored at an emission/excitation wavelength of 365/460 nm. The plate was read with a TECAN INFINITE 200 reader (Switzerland).

### 4.4. Annexin-V Assay

Apoptotic cells were evaluated with a Guava EasyCyte (Merck Millipore, MA, USA) flow cytometer using a fluorescein conjugated with Annexin V kit, following the manufacturer’s instructions. Annexin V and 7AAD dyes were used to analyze and identify apoptotic and non-apoptotic cells to cover a wide spectrum of cells. Annexin V (green) links to phosphatidylserine on the external membrane of apoptotic cells, while 7AAD (yellow) permeates and stains DNA in late-stage apoptotic and dead cells. Coloration enables the recognition of four cell populations: non-apoptotic cells (Annexin V− and 7AAD−), early apoptotic cells (annexin V+ and 7AAD−), late apoptotic cells (Annexin V+ and 7AAD+), and necrotic cells (Annexin V− and 7AAD+). In our experimental conditions, the early and late apoptotic cells were grouped together.

### 4.5. Lipid Droplet Size

Phase-contrast microscope pictures of differentiated cells were analyzed to determine the size of lipid droplets. The area of droplets was determined using Quantity One^®^ 1-D analysis software (Bio-Rad, Hercules, CA, USA). For each experimental condition, we determined the size of the lipid droplets in at least 100 cells from different microscope fields. The average areas were expressed in nm^2^.

### 4.6. Oil Red O Staining

Oil Red O staining is an indicator of intracellular lipid accumulation and was used to confirm adipogenic differentiation. The cell cultures were washed with PBS and then fixed with 10% formaldehyde for at least 1 h at RT. Then, the cells were washed with 60% isopropanol and left to dry completely. Subsequently, the cells were treated with Oil Red O staining solution for 10 min. After they were washed, the stained cultures were analyzed under a light microscope [47]. The percentage Oil red O staining-positive cells was calculated by counting a minimum of 300 cells in at least five different microscope fields. Initially, cells were identified by phase contrast microscopy (Leica DMi1-MC120HD, Leica Italia, Italy). Then, the same microscopic fields were analyzed by light microscopy (Leica DMi1-MC120HD, Leica Italia, Italy) to identify Oil red O stained cells. All reagents were obtained from Sigma-Aldrich (St. Louis, MO, USA) [47].

### 4.7. MitoTracker and UCP-1 Immunocytochemistry

MitoTracker^®^ (Invitrogen, Rome, Italy) was used to highlight mitochondria with 100 nmol/L probes in each cell culture; it accumulates in active mitochondria, due to its ability to pass across the plasma membrane through passive diffusion transport. Cell samples were incubated for 30 min in DMEM-free serum at 37 °C in the culture incubator. Then, the cells were washed with PBS. The experiment was carried out with two different analysis methods.

ICC analysis: The cells were fixed with 2% formaldehyde for 15 min at RT and washed with PBS. The UCP-1 level was detected in the cell cultures through antibodies against UCP-1 (Santa Cruz, TX, USA), according to the manufacturer’s instructions. Cell nuclei were stained with 4′,6-diamidino-2-phenylindole (DAPI) and then observed with a fluorescence microscope (Leica Italia, Italy). The percentage of UCP-1-positive cells was calculated by counting a minimum of 500 cells in different microscope fields.

FACS analysis: The cells were collected and analyzed with a Guava^®^ easyCyte™ flow cytometer (Merck Millipore, MA, USA) and analyzed using the easyCyte™ software.

### 4.8. RNA Extraction and RT-qPCR

RNA was extracted from cell cultures after drug treatment with TRIREAGENT (Molecular Research Center Inc., Cincinnati, OH, USA). To quantify the mRNA levels, we used NanoDrop Spectrophotometer (Thermo Scientifc, Waltham, MA, USA). To design primer pairs for RT-PCR reactions, we used OligoArchitect™ (Sigma-Aldrich, MO, USA); mRNA sequences were retrieved from a nucleotide data bank (National Center for Biotechnology Information). The appropriate region of GAPDH mRNA was used as a control (see Table 2 for primers list). Real-time PCR assays were carried out using LineGene 9600 Plus (BIOER, China); reactions were performed according to the manufacturer’s instructions. SYBR green PCR master mix (ABM, Italy) was used, and the 2-ΔΔCT method was employed as a relative quantification method [19].

### 4.9. Oxygen Consumption Assay

The oxygen consumption assay MitoXpress^®^ Xtra assay (see below) was performed on culture either in normal conditions or in media with only a specific energy substrate (glucose or fatty acids). In all the settings, cells were cultivated for 24 h in a substrate-limited medium containing DMEM (code A14430 from GIBCO LifeTech Monza, Italy), 1% FBS 0.5 mM glucose, 1 mM glutamax, and 0.5 mM carnitine. Then, the substrate-limited medium was replaced with the assay medium (PBS containing MgCl2 and CaCl2, 5 mM HEPES, 2.5 mM glucose, and 0.5 mM carnitine). After 60 min, the specific energy substrates (25 mM glucose or 200 μM palmitate) were added. Following 60 min-incubation we performed the oxygen consumption assay.

MitoXpress^®^ Xtra assay (Luxcel Biosciences, Dublin, Ireland) was used to evaluate the extracellular oxygen consumption rates (OCR). This assay uses an oxygen-sensing fluorophore that is quenched by O2 through molecular collision; therefore, the amount of fluorescence signal is inversely proportional to the amount of extracellular O2 in the sample. The first step was to measure the basal respiration, followed by the addition (in sequence) of oligomycin, FCCP (cyanide-p-trifluoromethox-yphenyl-hydrazon), and Antimycin A, whose job is to target the components of the electron transport chain (ETC) in the mitochondria to reveal key parameters of metabolic function. The oxygen employed by mitochondrial ATP production, maximal respiration, and non-mitochondrial respiration was determined by evaluating OCR in basal conditions and in the presence of the three compounds [12]. We determined the OCR according to the manufacturer’s instructions. The plate was read with a TECAN Infinite® 200 PRO (Männedorf, Switzerland).

### 4.10. Uptake and Release of Free Fatty Acids (FFA)

BODIPY FL C16 (Invitrogen, Waltham, MA, USA) is a green fluorescent fatty acid and was used to evaluate the FFA uptake was performed according to Capasso et al. [48]. In details, cells were serum starved for 16 h and then incubated in DMEM FluroBrite (Gibco) with 10 µM of BODIPY FL C16 for 2 h. Then, cells were washed three times with PBS and incubated for different time periods with a complete medium (D-MEM containing 10% FBS without BODIPY) for 2–4 h. The plate was read at different times, as indicated with the TECAN INFINITE 200 (Männedorf, Switzerland).

The release of fatty acids was evaluated according to Meisner and Tenney, with some modifications [49]. In brief, cells were incubated in KREBS buffer containing 2% BSA for 10 min. Then, 10 min and 30 min following treatment with 0.5 µg/mL epinephrine, aliquots of culture media were collected, and the pH was determined with a SevenMulti pH meter S-40 Mettler Toledo (Sigma-Aldrich, St. Louis, MO, USA).

### 4.11. Western Blot

Cells were lysed in a buffer containing 0.1% Triton (Bio-Rad, Hercules, CA, USA) for 30 min in ice; 20 μg of each lysate was electrophoresed in a polyacrylamide gel and electroblotted into a nitrocellulose membrane using Biorad transblot apparatus. We used the following primary antibodies: UCP1 (sc518024, Santa Cruz, USA), LPL (MABS1270), and GAPDH (G8795) from Sigma-Aldrich (St. Louis, MO, USA), PPARG (PA3-821A) from Thermo Fischer Scientific (Waltham, Massachusetts, USA). The primary antibodies were added to a nitrocellulose membrane in a buffer solution (TTBS 0.01X and 3% free fat milk) and incubated overnight at 4 °C. Then, the membranes were washed three times in T-TBS 1X and incubated with a horseradish peroxidase-conjugated secondary antibody (ImmunoReagents, Raleigh, NC, USA) and reacted with ECL plus reagent (Merck Millipore, Burlington, MA, USA). All antibodies were used according to the manufacturer’s instructions. The mean value was quantified densitometrically using Quantity One^®^ 1-D analysis software (Bio-Rad, Hercules, CA, USA).

### 4.12. Statistical Analysis

Statistical significance was evaluated with ANOVA, post-hoc test Tukey test followed by Student’s *t*-test or Bonferroni’s test. Experiments were carried out three times on three biological replicates. A mixed-model variance analysis was used for data with continuous outcomes. Data were evaluated with a GraphPad Prism version 8 statistical software package (GraphPad, La Jolla, CA, USA).

## 5. Conclusions

In conclusion, we found that the tested drugs, in particular irisin, are promising candidates for browning as a valid therapy for counteracting obesity. Our future research will examine accurate in vivo treatment on the browning effect of these drugs, in addition to a painstaking investigation of the potential side effects.

## Figures and Tables

**Table 1:** Cell viability after 3 days of incubation with drugs, at different concentrations. For each drug, the last column indicates the concentration used in all the described experiments.

	Drug	1 nM	10 nM	100 nM	1 µM	10 µM	100 µM	Working Concentration
**Alive cells**	**GW**	100%	100%	100%	100%	99%	94%	**1 nM**
	**SID**	100%	100%	100%	100%	98%	73%	**1 nM**
	**ROS**	100%	100%	100%	97%	74%	58%	**43 nM**
	**IRI**	100%	100%	100%	---	---	---	**20 nM**

**Table 2:** RT-qPCR primers list.

Gene	Sequence	Annealing T (°C)
**GAPDH**	5′ GGAGTCAACGGATTTGGTCGT 3′ 5′ ACGGTGCCATGGAATTTGC 3′	58
**UCP1**	5′ TACAGAATAATAGCAACAAC 3′ 5′ CCTCCTTCATTAGATCATAT 3′	55
**PPARG**	5′ TCGACCACGTCAATCCAGAGT 3′ 5′ TCGCCTTTGCTTTGGTCAG 3′	60
**CEBPA**	5′ GCCGACGGAGAGTCTTATT 3′ 5′ CITGTGCATGTTGAATGTG 3′	61
**CEBPB**	5′ AACATGGCIGAACGCGIGT 3′ 5′ TCACAGCACAGCCCGT 3′	60
**LPL**	5′ ATGGCIGGACGGTAACAGGAA 3′ 5′ TGACAGCCAGTCCACCACAAT 3′	60

## Data Availability

Not applicable.

## References

[1] Nguyen N.T., Varela J.E. (2017). Bariatric surgery for obesity and metabolic disorders: State of the art. Nat. Rev. Gastroenterol. Hepatol..

[2] Stimac D., Klobucar Majanovic S., Belancic A. (2020). Endoscopic Treatment of Obesity: From Past to Future. Dig. Dis..

[3] Jakus P.B., Sandor A., Janaky T., Farkas V. (2008). Cooperation between BAT and WAT of rats in thermogenesis in response to cold, and the mechanism of glycogen accumulation in BAT during reacclimation. J. Lipid. Res..

[4] Lidell M.E. (2019). Brown Adipose Tissue in Human Infants. Handb. Exp. Pharmacol..

[5] Dawkins M.J., Scopes J.W. (1965). Non-shivering thermogenesis and brown adipose tissue in the human new-born infant. Nature.

[6] Cypess A.M., Lehman S., Williams G., Tal I., Rodman D., Goldfine A.B., Kuo F.C., Palmer E.L., Tseng Y.H., Doria A. (2009). Identification and importance of brown adipose tissue in adult humans. N. Engl. J. Med..

[7] van Marken Lichtenbelt W.D., Vanhommerig J.W., Smulders N.M., Drossaerts J.M., Kemerink G.J., Bouvy N.D., Schrauwen P., Teule G.J. (2009). Cold-activated brown adipose tissue in healthy men. N. Engl. J. Med..

[8] Virtanen K.A., Lidell M.E., Orava J., Heglind M., Westergren R., Niemi T., Taittonen M., Laine J., Savisto N.J., Enerback S. (2009). Functional brown adipose tissue in healthy adults. N. Engl. J. Med..

[9] Choi Y., Yu L. (2021). Natural Bioactive Compounds as Potential Browning Agents in White Adipose Tissue. Pharm. Res..

[10] Wankhade U.D., Shen M., Yadav H., Thakali K.M. (2016). Novel Browning Agents, Mechanisms, and Therapeutic Potentials of Brown Adipose Tissue. Biomed. Res. Int..

[11] Rosenwald M., Wolfrum C. (2014). The origin and definition of brite versus white and classical brown adipocytes. Adipocyte.

[12] Tamucci K.A., Namwanje M., Fan L., Qiang L. (2018). The dark side of browning. Protein Cell.

[13] Bargut T.C.L., Souza-Mello V., Aguila M.B., Mandarim-de-Lacerda C.A. (2017). Browning of white adipose tissue: Lessons from experimental models. Horm. Mol. Biol. Clin. Investig..

[14] Spiegelman B.M. (2013). Banting Lecture 2012: Regulation of adipogenesis: Toward new therapeutics for metabolic disease. Diabetes.

[15] Barbatelli G., Murano I., Madsen L., Hao Q., Jimenez M., Kristiansen K., Giacobino J.P., De Matteis R., Cinti S. (2010). The emergence of cold-induced brown adipocytes in mouse white fat depots is determined predominantly by white to brown adipocyte transdifferentiation. Am. J. Physiol. Endocrinol. Metab..

[16] Li S., Li Y., Xiang L., Dong J., Liu M., Xiang G. (2018). Sildenafil induces browning of subcutaneous white adipose tissue in overweight adults. Metabolism.

[17] Moisan A., Lee Y.K., Zhang J.D., Hudak C.S., Meyer C.A., Prummer M., Zoffmann S., Truong H.H., Ebeling M., Kiialainen A. (2015). White-to-brown metabolic conversion of human adipocytes by JAK inhibition. Nat. Cell Biol..

[18] Morganstein D.L., Wu P., Mane M.R., Fisk N.M., White R., Parker M.G. (2010). Human fetal mesenchymal stem cells differentiate into brown and white adipocytes: A role for ERRalpha in human UCP1 expression. Cell Res..

[19] Di Maio G., Alessio N., Demirsoy I.H., Peluso G., Perrotta S., Monda M., Di Bernardo G. (2021). Evaluation of Browning Agents on the White Adipogenesis of Bone Marrow Mesenchymal Stromal Cells: A Contribution to Fighting Obesity. Cells.

[20] Galderisi U., Peluso G., Di Bernardo G. (2021). Clinical Trials Based on Mesenchymal Stromal Cells are Exponentially Increasing: Where are We in Recent Years?. Stem Cell Rev. Rep..

[21] Squillaro T., Peluso G., Galderisi U., Di Bernardo G. (2020). Long non-coding RNAs in regulation of adipogenesis and adipose tissue function. Elife.

[22] Bostrom P., Wu J., Jedrychowski M.P., Korde A., Ye L., Lo J.C., Rasbach K.A., Bostrom E.A., Choi J.H., Long J.Z. (2012). A PGC1-alpha-dependent myokine that drives brown-fat-like development of white fat and thermogenesis. Nature.

[23] Bhagavathula N., Nerusu K.C., Lal A., Ellis C.N., Chittiboyina A., Avery M.A., Ho C.I., Benson S.C., Pershadsingh H.A., Kurtz T.W. (2004). Rosiglitazone inhibits proliferation, motility, and matrix metalloproteinase production in keratinocytes. J. Invest. Dermatol..

[24] Erdogan A., Luedders D.W., Muenz B.M., Schaefer C.A., Tillmanns H., Wiecha J., Kuhlmann C.R. (2007). Sildenafil inhibits the proliferation of cultured human endothelial cells. Int. J. Biomed. Sci..

[25] Pechery A., Fauconnet S., Bittard H., Lascombe I. (2016). Apoptotic effect of the selective PPARbeta/delta agonist GW501516 in invasive bladder cancer cells. Tumour. Biol..

[26] Ye W., Wang J., Lin D., Ding Z. (2020). The immunomodulatory role of irisin on osteogenesis via AMPK-mediated macrophage polarization. Int. J. Biol. Macromol..

[27] Lim J., Park H.S., Kim J., Jang Y.J., Kim J.H., Lee Y., Heo Y. (2020). Depot-specific UCP1 expression in human white adipose tissue and its association with obesity-related markers. Int. J. Obes..

[28] Nedergaard J., Cannon B. (2014). The browning of white adipose tissue: Some burning issues. Cell Metab..

[29] De Pauw A., Tejerina S., Raes M., Keijer J., Arnould T. (2009). Mitochondrial (dys)function in adipocyte (de)differentiation and systemic metabolic alterations. Am. J. Pathol..

[30] Forni M.F., Peloggia J., Trudeau K., Shirihai O., Kowaltowski A.J. (2016). Murine Mesenchymal Stem Cell Commitment to Differentiation Is Regulated by Mitochondrial Dynamics. Stem Cells.

[31] Lee J.H., Park A., Oh K.J., Lee S.C., Kim W.K., Bae K.H. (2019). The Role of Adipose Tissue Mitochondria: Regulation of Mitochondrial Function for the Treatment of Metabolic Diseases. Int. J. Mol. Sci..

[32] Abou-Rjeileh U., Contreras G.A. (2021). Redox Regulation of Lipid Mobilization in Adipose Tissues. Antioxidants.

[33] Hankir M.K., Klingenspor M. (2018). Brown adipocyte glucose metabolism: A heated subject. EMBO Rep..

[34] Wade G., McGahee A., Ntambi J.M., Simcox J. (2021). Lipid Transport in Brown Adipocyte Thermogenesis. Front. Physiol..

[35] Bonet M.L., Oliver P., Palou A. (2013). Pharmacological and nutritional agents promoting browning of white adipose tissue. Biochim Biophys. Acta.

[36] Chen Y.C., Yu Y.H. (2018). The potential of brown adipogenesis and browning in porcine bone marrow-derived mesenchymal stem cells1. J. Anim. Sci..

[37] Polyzos S.A., Anastasilakis A.D., Efstathiadou Z.A., Makras P., Perakakis N., Kountouras J., Mantzoros C.S. (2018). Irisin in metabolic diseases. Endocrine.

[38] Zhang Y., Li R., Meng Y., Li S., Donelan W., Zhao Y., Qi L., Zhang M., Wang X., Cui T. (2014). Irisin stimulates browning of white adipocytes through mitogen-activated protein kinase p38 MAP kinase and ERK MAP kinase signaling. Diabetes.

[39] Zhang Y., Liu Q., Yu J., Yu S., Wang J., Qiang L., Gu Z. (2017). Locally Induced Adipose Tissue Browning by Microneedle Patch for Obesity Treatment. ACS Nano.

[40] Norheim F., Langleite T.M., Hjorth M., Holen T., Kielland A., Stadheim H.K., Gulseth H.L., Birkeland K.I., Jensen J., Drevon C.A. (2014). The effects of acute and chronic exercise on PGC-1alpha, irisin and browning of subcutaneous adipose tissue in humans. FEBS J..

[41] Fernyhough M.E., Vierck J.L., Hausman G.J., Mir P.S., Okine E.K., Dodson M.V. (2004). Primary adipocyte culture: Adipocyte purification methods may lead to a new understanding of adipose tissue growth and development. Cytotechnology.

[42] Macotela Y., Emanuelli B., Mori M.A., Gesta S., Schulz T.J., Tseng Y.H., Kahn C.R. (2012). Intrinsic differences in adipocyte precursor cells from different white fat depots. Diabetes.

[43] Martinez-Santibanez G., Cho K.W., Lumeng C.N. (2014). Imaging white adipose tissue with confocal microscopy. Methods Enzymol..

[44] McNeill B.T., Morton N.M., Stimson R.H. (2020). Substrate Utilization by Brown Adipose Tissue: What’s Hot and What’s Not?. Front. Endocrinol..

[45] Santos A.I., Carreira B.P., Nobre R.J., Carvalho C.M., Araujo I.M. (2014). Stimulation of neural stem cell proliferation by inhibition of phosphodiesterase 5. Stem Cells Int..

[46] Deng J., Zhang N., Wang Y., Yang C., Wang Y., Xin C., Zhao J., Jin Z., Cao F., Zhang Z. (2020). FNDC5/irisin improves the therapeutic efficacy of bone marrow-derived mesenchymal stem cells for myocardial infarction. Stem Cell Res. Ther..

[47] Alessio N., Pipino C., Mandatori D., Di Tomo P., Ferone A., Marchiso M., Melone M.A.B., Peluso G., Pandolfi A., Galderisi U. (2018). Mesenchymal stromal cells from amniotic fluid are less prone to senescence compared to those obtained from bone marrow: An in vitro study. J. Cell Physiol..

[48] Capasso S., Alessio N., Di Bernardo G., Cipollaro M., Melone M.A., Peluso G., Giordano A., Galderisi U. (2014). Silencing of RB1 and RB2/P130 during adipogenesis of bone marrow stromal cells results in dysregulated differentiation. Cell Cycle.

[49] Meisner H., Tenney K. (1977). pH as an indicator of free fatty acid release from adipocytes. J. Lipid. Res..

